# High-flow nasal cannula oxygen therapy versus non-invasive ventilation for chronic obstructive pulmonary disease patients after extubation: a multicenter, randomized controlled trial

**DOI:** 10.1186/s13054-020-03214-9

**Published:** 2020-08-06

**Authors:** Dingyu Tan, Joseph Harold Walline, Bingyu Ling, Yan Xu, Jiayan Sun, Bingxia Wang, Xueqin Shan, Yunyun Wang, Peng Cao, Qingcheng Zhu, Ping Geng, Jun Xu

**Affiliations:** 1 Department of Emergency Medicine, Clinical Medical College of Yangzhou University, Northern Jiangsu People’s Hospital, Yangzhou, 225001 China; 2 Accident and Emergency Medicine Academic Unit, The Chinese University of Hong Kong, Prince of Wales Hospital, Hong Kong SAR, China; 3 Pharmacy Department, Clinical Medical College of Yangzhou University, Northern Jiangsu People’s Hospital, Yangzhou, 225001 China; 4 Intensive Care Unit, Yangzhou Hongquan Hospital, Yangzhou, 225200 China; 5 Emergency Department, Peking Union Medical College Hospital, Chinese Academy of Medical Sciences, Beijing, 100730 China

**Keywords:** Chronic obstructive pulmonary diseases, Respiratory failure, High-flow nasal cannula, Non-invasive ventilation, Pulmonary infection control window, Hypercapnia

## Abstract

**Background:**

High-flow nasal cannula (HFNC) oxygen therapy is being increasingly used to prevent post-extubation hypoxemic respiratory failure and reintubation. However, evidence to support the use of HFNC in chronic obstructive pulmonary disease (COPD) patients with hypercapnic respiratory failure after extubation is limited. This study was conducted to test if HFNC is non-inferior to non-invasive ventilation (NIV) in preventing post-extubation treatment failure in COPD patients previously intubated for hypercapnic respiratory failure.

**Methods:**

COPD patients with hypercapnic respiratory failure who were already receiving invasive ventilation were randomized to HFNC or NIV at extubation at two large tertiary academic teaching hospitals. The primary endpoint was treatment failure, defined as either resumption of invasive ventilation or switching to the other study treatment modality (NIV for patients in the NFNC group or vice versa).

**Results:**

Ninety-six patients were randomly assigned to the HFNC group or NIV group. After secondary exclusion, 44 patients in the HFNC group and 42 patients in the NIV group were included in the analysis. The treatment failure rate in the HFNC group was 22.7% and 28.6% in the NIV group—risk difference of − 5.8% (95% CI, − 23.8–12.4%, *p* = 0.535), which was significantly lower than the non-inferior margin of 9%. Analysis of the causes of treatment failure showed that treatment intolerance in the HFNC group was significantly lower than that in the NIV group, with a risk difference of − 50.0% (95% CI, − 74.6 to − 12.9%, *p* = 0.015). One hour after extubation, the mean respiratory rates of both groups were faster than their baseline levels before extubation (*p* < 0.050). Twenty-four hours after extubation, the respiratory rate of the HFNC group had returned to baseline, but the NIV group was still higher than the baseline. Forty-eight hours after extubation, the respiratory rates of both groups were not significantly different from the baseline. The average number of daily airway care interventions in the NIV group was 7 (5–9.3), which was significantly higher than 6 (4–7) times in the HFNC group (*p* = 0.006). The comfort score and incidence of nasal and facial skin breakdown of the HFNC group was also significantly better than that of the NIV group [7 (6–8) vs 5 (4–7), *P* < 0.001] and [0 vs 9.6%, *p* = 0.027], respectively.

**Conclusion:**

Among COPD patients with severe hypercapnic respiratory failure who received invasive ventilation, the use of HFNC after extubation did not result in increased rates of treatment failure compared with NIV. HFNC also had better tolerance and comfort than NIV.

**Trial registration:**

chictr.org (ChiCTR1800018530). Registered on 22 September 2018, http://www.chictr.org.cn/usercenter.aspx

## Introduction

Chronic obstructive pulmonary disease (COPD) is one of the leading causes of death worldwide. Acute hypercapnic respiratory failure is a common serious complication of COPD, and invasive mechanical ventilation is often required for severe cases. Longer durations of invasive mechanical ventilation will increase the incidence of ventilator-associated pneumonia and difficulty weaning off ventilation [1, 2]. Multiple studies have shown that a sequential strategy with non-invasive ventilation (NIV) using a pulmonary infection control (PIC) window as the switching point can reduce the duration of invasive ventilation in COPD patients and significantly improve prognosis [3, 4].

The success of NIV is closely related to the experience and abilities of the treating medical staff, the level of education and compliance of patients, and the performance of the NIV device [5, 6]. Due primarily to poor patient tolerance, NIV fails in approximately 15 to 25% of patients, potentially leading to endotracheal intubation [7–9]. For post-extubation patients with COPD who cannot tolerate NIV or have contraindications to NIV, alternative respiratory support methods are urgently needed.

High-flow nasal cannula (HFNC) oxygen therapy is a new type of respiratory support system which can supply high flow mixed gases through special nasal prongs at a sufficient temperature and humidity for patient comfort. Many studies have confirmed that the comfort and tolerance of HFNC is significantly higher than that of NIV [10–12]. As an alternative to NIV, HFNC has been shown to be as efficacious as NIV in preventing post-extubation respiratory failure or re-intubation in patients with hypoxemic respiratory failure [13, 14]. However, the post-extubation application of HFNC in COPD patients with hypercapnic respiratory failure has not been widely studied. In a pilot study, HFNC was reported to maintain similar patient vital signs and arterial blood gases as NIV in post-extubated hypercapnic COPD patients [15].

This trial was conducted to test the hypothesis that HFNC immediately after extubation is non-inferior to NIV in reducing treatment failure in COPD patients previously intubated for hypercapnic respiratory failure.

## Materials and methods

### Study design and ethical approval

This was a multicenter, unblinded, non-inferiority, randomized controlled trial, registered at chictr.org (ChiCTR1800018530). From January 2019 to February 2020, the study was performed in the intensive care units (ICUs) of two large tertiary-care hospitals. This study was approved by the human subjects ethics committees of the two hospitals involved (2018KY-081 and 20180012), and informed consent was obtained from all enrolled patients or their relatives.

### Screening of patients

COPD patients with hypercapnic respiratory failure who received invasive ventilation were screened for enrollment. The diagnosis of COPD was established according to the GOLD criteria [16]. Other inclusion criteria included patients who were ≤ 85 years of age, able to care for themselves within the past year, respiratory failure induced by broncho-pulmonary infection, and meeting criteria of the PIC window. Exclusion criteria were age less than 18 years; lacking informed consent; contraindications to NIV (oral or facial trauma, poor sputum excretion ability, hemodynamic instability); poor short-term prognosis (high risk of death within7 days or receiving palliative treatment); heart, brain, liver, or kidney failure; tracheotomy; or a weak cough ability during the PIC window.

The following types of patients were secondarily excluded: withdrawn informed consent, loss to follow-up, uncertain 28-day survival, discharge from hospital within 48 h after enrollment, and patients who refused to use their assigned device.

### Experimental procedure

The settings of the enrolled patients’ invasive mechanical ventilation were adjusted by the attending physician according to the patient’s ventilation status and blood gas analysis. The patients were randomly divided into the HFNC group and the NIV group when the PIC window appeared. Randomization was performed using a computer-generated random number generator, and allocation was concealed through an opaque envelope. These envelopes were kept in permuted blocks of ten, five each for NIV and HFNC, to ensure an even distribution of subject numbers in both groups at both centers.

All subjects receiving NIV (Philips V60 or BiPap Vision) were set in S/T mode with a standard oral-nasal (full-face) mask (RT040). NIV settings were adjusted with an adaptive method: the initial expiratory pressure airway pressure was set to 4 cmH_2_O, and the pressure level was gradually increased to ensure that the patient could trigger the NIV device with each inhalation. The inspiratory airway pressure was initially set to 8 cmH_2_O and gradually increased to achieve a satisfactory tidal volume with acceptable tolerance. The pressure level and the fraction of inspiration oxygen (FiO_2_) were adjusted to maintain a respiratory rate ≤ 28/min, a pulse oxygen saturation (SpO_2_) of 88–92%, and a partial pressure of arterial carbon dioxide (PaCO_2_) of either 45–60 mmHg or the last PaCO_2_ level recorded prior to extubation.

Subjects randomized to the HFNC group (AIRVO™ 2, Fisher & Paykel Healthcare, Auckland, New Zealand) were given suitable large-bore nasal prongs selected according to the size of the patients’ nostrils. The initial airflow was set at 50 L/min and adjusted according to patient tolerance. The HFNC was set to an absolute humidity of 44 mg H_2_O/L, temperature was set to 37 °C, and FiO_2_ was adjusted to maintain an SpO_2_ of 88–92%.

The patient’s initial respiratory support was targeted to last at least 2 h and then continued as needed. Nasal cannula oxygen was administered during any interruptions to NIV. NIV or HFNC were discontinued when the total daily treatment duration was less than 4 h and could be reused if needed. Treatment success was defined as no need for respiratory support within 72 h after stopping NIV or HFNC.

### Outcomes

The primary outcome was treatment failure, defined as a return to invasive mechanical ventilation, or a switch in respiratory support modality (i.e., changing from HFNC to NIV or from NIV to HFNC). Secondary outcomes included arterial blood gas analysis [pH, PaO_2_ (partial pressure of oxygen in arterial blood), PaCO_2_, and FiO_2_] and vital signs such as respiratory rate, heart rate, and blood pressure at 1, 24, and 48 h after extubation, as well as the total duration of respiratory support after extubation, the daily number of nursing airway care interventions, the patients’ comfort score, the patients’ dyspnea score, the incidence of nasofacial skin breakdown, 28-day mortality, and total ICU and hospital lengths of stay.

Airway care interventions were defined as the need for nursing staff to correct unplanned device displacement due to intolerance, discomfort, or another reason, or the need for nursing staff to assist in the removal or fixation of the device due to sputum, eating, or drinking. The patient’s comfort score was assessed using a modified 10-cm visual analog scale, in which 1 meant very uncomfortable and 10 meant very comfortable [11]. The patients’ dyspnea was evaluated with a Borg rating scale [17]. The criteria for reintubation in this study were [18, 19] cardiac arrest or obvious hemodynamic instability, refractory hypoxemia (PaO_2_ < 50 mmHg with sufficient oxygen therapy), significant hypercapnia with pH ≤ 7.20, severe disturbances of consciousness such as coma, respiratory depression (respiratory frequency < 8/min), or severe dyspnea (respiratory frequency > 40/min).

### Sample size and statistical analysis

Based on previous studies [20, 21], we estimated that NIV would fail in 22% patients (either intubation or intolerance) of included COPD patients, and the absolute difference of treatment failure rates between HFNC and NIV was likely to fall between 4 and 12% [14]. After discussions with three senior pulmonologists, we set the non-inferiority cutoff at 9%. To assess non-inferiority using an α = 0.50, β = 0.20, and 1-sided testing, 44 subjects were needed in each group (88 total).

For the primary outcome, analysis was performed both on an intention-to-treat and on a per-protocol basis. The Kaplan-Meier method was used to draw the cumulative survival and failure curves. The Kolmogorov-Smirnov test was used to test the normal distribution for measurement data. Normally distributed data were expressed as means ± standard deviation, and the skewed distributed data was reported as medians with interquartile (25th–75th) percentiles. The two groups were compared using *t* tests or Mann-Whitney *U* tests. Numeric data were expressed as a percentage (%), using *χ*^2^ or Fisher’s exact probability tests. The comparison of vital signs and blood gas analyses at multiple time points was performed by repeated measures analysis of variance, or non-parametric test of multiple correlated samples (Friedman test for heterogeneity of variance or the skewed distributed data), in which the significance level was adjusted using the Bonferroni correction method. All data analysis was conducted using SPSS 26.0 (IBM Corporation, Armonk, NY, USA).

## Results

### Patient characteristics

**Fig. 1:**
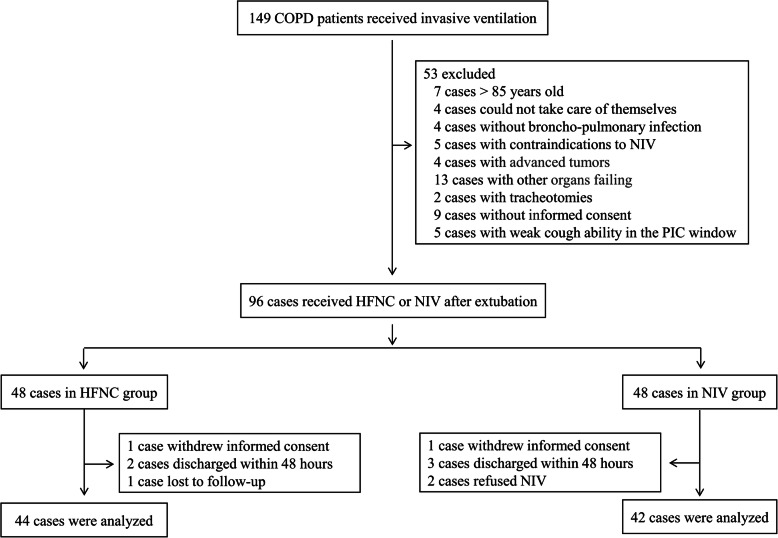
Flow chart of patient enrollment. COPD, chronic obstructive pulmonary disease; ICU, intensive care unit; HFNC, high-flow nasal cannula oxygen therapy; NIV, non-invasive ventilation; PIC, pulmonary infection control

Among 149 COPD patients who received invasive ventilation in our enrolling centers during the study period, 96 (64.4%) patients were randomized to the NIV or HFNC groups after 53 patients were excluded for various reasons (see Fig. 1). Six patients in the NIV group and four patients in the HFNC group were secondarily excluded. Finally, 42 patients in the NIV group and 44 patients in the HFNC group were included in the analysis. Demographic, relevant comorbidities, smoking history, COPD medications, respiratory therapy at home, available pulmonary function tests, the Simplified Acute Physiology Score II (SAPS II), and the Acute Physiological and Chronic Health Status Score II (APACHE II) at admission in the two groups were similar (see Table 1). Seventeen (38.6%) patients in the HFNC group and 18 (42.9%) in the NIV group initially received NIV or HFNC after admission before invasive ventilation, and the remaining patients received invasive ventilation directly. There were also no significant differences in respiratory parameters, blood gas analyses, and vital signs between the two groups at the time of enrollment (PIC window before extubation). The stable FiO_2_ after extubation in the HFNC group was 0.32 (0.28–0.38), which was not significantly different from 0.35 (0.30–0.40) in the NIV group.

**Table 1:** Characteristics of enrolled patients

Characteristics	HFNC (*n* = 44)	NIV (*n* = 42)	*p* value
Male, *n* (%)	27 (64.3)	23 (55.6)	0.259
Age, years	68.4 ± 9.3	71.4 ± 7.8	0.107
History of COPD, years	8 (5.3–10)	10 (6–12.3)	0.063
Smoking history, *n* (%)	20 (45.5)	26 (61.9)	0.126
Current	8 (45.5)	11 (45.5)	0.371
Comorbidities, *n* (%)			
Diabetes mellitus	10 (22.7)	14 (33.3)	0.273
Hypertension	25 (56.8)	17 (40.5)	0.130
Coronary artery disease	13 (29.5)	16 (38.1)	0.402
Chronic liver disease	2 (4.5)	5 (11.9)	0.260
Chronic kidney disease	12 (27.3)	8 (19.0)	0.367
Cerebrovascular disease	5 (11.4)	7 (16.7)	0.545
Malignancy	6 (13.6)	3 (7.1)	0.325
Medication before exacerbation, *n* (%)			
Inhaled corticosteroids	11 (30.8)	15 (34.9)	0.280
Beta adrenoceptor agonist	17 (38.6)	11 (26.2)	0.218
Anticholinergics	5 (11.4)	8 (19.0)	0.320
Home oxygen therapy, *n* (%)			
NCO	8 (18.2)	5 (11.9)	0.417
NIV	5 (11.4)	7 (16.7)	0.478
Lung function test before exacerbation (*n* = 52)*			
FEV_1_, %	41.3 ± 11.0	43.2 ± 12.6	0.577
FEV_1_/FVC, %	40.9 ± 9.8	42.4 ± 9.6	0.580
APACHE II score	14 (11–18.8)	13 (10.8–16)	0.323
SAPS II score	27 (22–32.8)	30 (24–34.8)	0.138
NIV or HFNC before IMV, *n* (%)			
NIV	10 (22.7)	13 (31.0)	0.389
HFNC	7 (15.9)	5 (11.9)	0.592
Characteristics at the PIC window			
Pressure support, cmH_2_O	11.7 ± 2.0	11.5 ± 1.7	0.584
PEEP, cmH_2_O	5.5 (5–6)	6 (5–6)	0.512
Tidal volume, mL	442.7 ± 57.7	431.2 ± 51.9	0.333
Minute volume, L/min	8.5 ± 2.0	9.1 ± 2.5	0.292
Respiratory frequency, /min	18 (16–23)	21 (16–26)	0.158
Arterial pH	7.48 (7.42–7.51)	7.45 (7.40–7.49)	0.102
PaCO_2_, mmHg	50.5 (48–57.8)	53 (48.8–61.3)	0.236
PaO_2_/FiO_2_, mmHg	239.2 ± 47.0	229.3 ± 42.0	0.307
Heart rate, beats/min	92 (80.5–107.8)	87.5 (80.8–101.5)	0.362
Mean arterial pressure, mmHg	88 ± 6.0	84.5 ± 10.3	0.057
Duration of IMV, hours	98 (65.8–175.8)	114 (79.3–147.5)	0.792
FiO_2_ after extubation (stable)	0.32 (0.28–0.38)	0.35 (0.30–0.40)	0.447

Data are shown as means ± standard deviation, number (%) patients, or median (interquartile range)

*HFNC* high-flow nasal cannula oxygen therapy, *NIV* non-invasive ventilation, *COPD* chronic obstructive pulmonary disease, *NCO* nasal cannula oxygen, *ICU* intensive care unit, *APACHE II* Acute Physiology and Chronic Health Evaluation II, *SAPS II* Simplified Acute Physiology Score II, *IMV* invasive mechanical ventilation, *PIC* pulmonary infection control, *PEEP* positive end expiratory pressure, *PaCO*_*2*_ partial pressure of arterial carbon dioxide, *PaO*_*2*_ partial pressure of arterial oxygen, *FiO*_*2*_ fraction of inspiration oxygen

*Twenty-five cases in the HFNC group and 27 cases in the NIV group

### Primary outcome

**Fig. 2:**
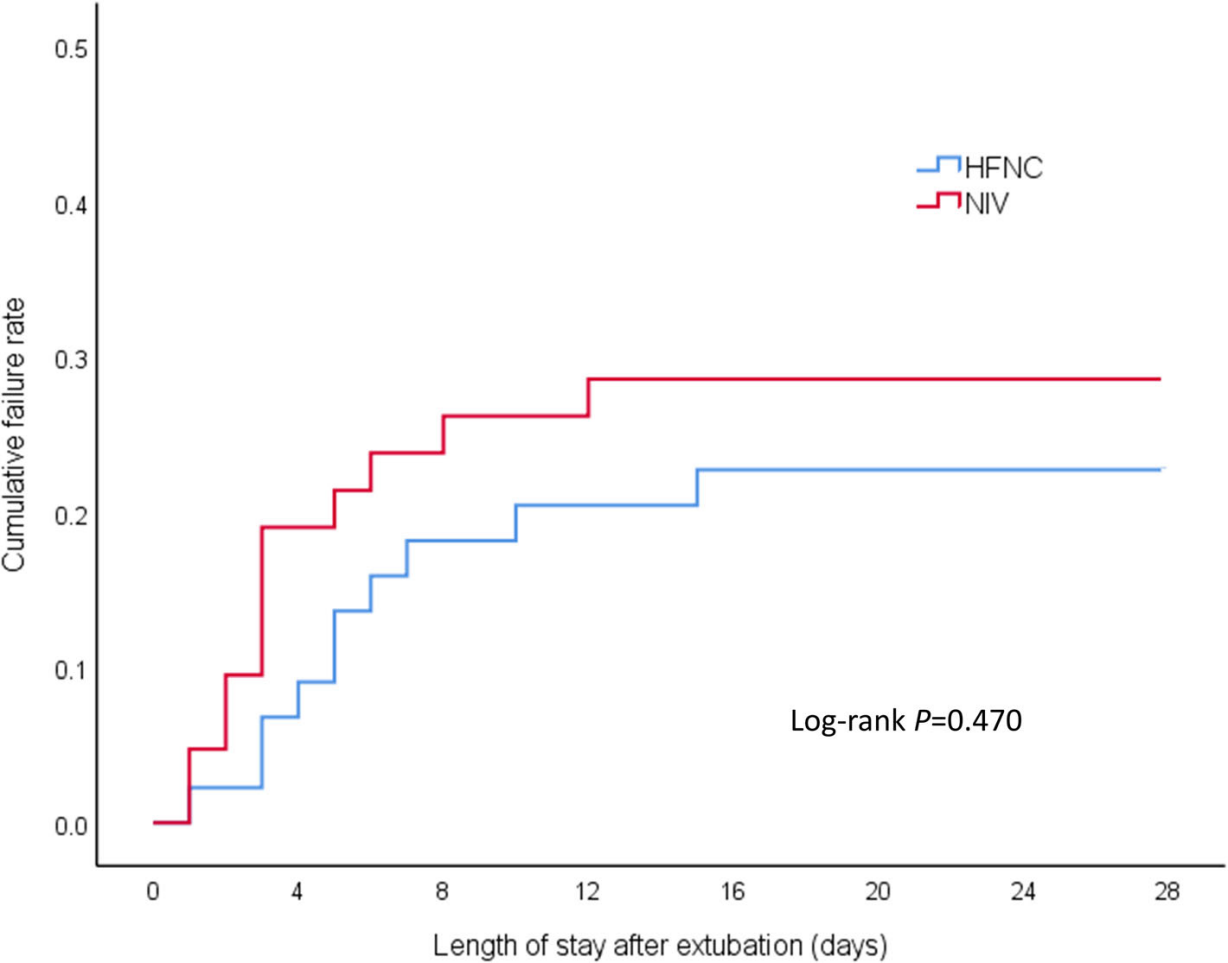
Kaplan-Meier curve analysis for cumulative failure rate. HFNC, high-flow nasal cannula oxygen therapy; NIV, non-invasive ventilation

Treatment failure occurred in 10 patients (22.7%) in the HFNC group and 12 patients (28.6%) in the NIV group (risk difference, − 5.8%; 95% CI, − 23.8 to 12.4%; see Table 2). Additionally, Kaplan-Meier curves showed no statistical difference in cumulative failure rates between the two groups (log-rank test 0.521, *p* = 0.470, see Fig. 2). Among the patients with treatment failure, the intubation rate in the HFNC group was similar to that of the NIV group (− 0.65%; 95% CI, − 16.01 to 14.46%), and the treatment switch rate was lower than that in the NIV group (− 5.2%; 95% CI, − 19.82 to 9.05%). However, there were no significant differences between the two groups in intubation or treatment switch rate.

**Table 2:** Primary outcome and cause analysis

	HFNC (*n* = 44)	NIV (*n* = 42)	Risk difference, % (95% CI)	*p* value
Primary outcome, *n* (%)				
Treatment failure	10 (22.7)	12 (28.6)	− 5.8 (− 23.8 to 12.4)	0.535
Invasive ventilation	6 (13.6)	6 (14.29)	− 0.65 (− 16.01 to 14.46)	0.931
Treatment switch	4 (9.1)	6 (14.3)	− 5.2 (− 19.82 to 9.05)	0.516
Analysis of treatment failure, *n* (%)				
Treatment intolerance	0/10 (0)	6/12 (50.0)	− 50.0 (− 74.62 to − 12.9)	0.015
Aggravation of respiratory distress	5/10 (50)	2/12 (16.67)	33.33 (− 5.21 to 62.27)	0.172
Aggravation of hypoxemia	2/10 (20)	1/12 (8.33)	11.67 (− 18.95 to 43.4)	0.571
Aggravation of carbon dioxide retention	3/10 (30)	3/12 (25)	5 (− 29.15 to 39.33)	1.0

*HFNC* high-flow nasal cannula oxygen therapy, *NIV* non-invasive ventilation

Analysis of the causes of treatment failure showed that treatment intolerance was significantly lower in the HFNC group than in the NIV group, with a risk difference of − 50.0% (95% CI, − 74.6 to − 12.9%, *p* = 0.015, see Table 2). There was no significant difference between the two groups in exacerbated respiratory distress, hypoxemia, or carbon dioxide retention. The causes for six intolerances in the NIV group were feelings of claustrophobia (*n* = 2), excessive air flow or pressure (*n* = 2), breathlessness (*n* = 1), and headache (*n* = 1).

### Secondary outcomes

#### Vital signs and blood gas analyses

Heart rate and mean arterial pressure within 48 h after extubation in the two groups were not significantly different from baseline levels before extubation. Respiratory rate in both groups was faster than before extubation at 1 h after extubation (*p* < 0.050, see Table 3). The respiratory rate 24 h after extubation in the HFNC group had decreased to its baseline and was lower than the respiratory rate in the NIV group [20 (17.3–24.5)/min vs 24.5 (18–27)/min, *p* < 0.050]. The NIV group’s respiratory rate was also higher than its baseline level. There was no significant difference in respiratory rate between the two groups at 48 h after extubation.

**Table 3:** Vital signs and arterial blood gas analysis

	HR (bpm)	MAP (mmHg)	RR (bpm)	pH	PaCO_2_ (mmHg)	PaO_2_/FiO_2_ (mmHg)
NIV (*n* = 42)						
Baseline	87.5 (80.8–101.5)	84.5 ± 10.3	21 (16–26)	7.45 (7.40–7.49)	53 (48.8–61.3)	229.3 ± 42.0
1 h	90.5 (80–108.3)	86.8 ± 10.2	25.5 (18–28)*	7.44 (7.39–7.48)	55 (48.8–61.3)	218.9 ± 37.8*
24 h	94 (79.8–105.3)	87.6 ± 11.1	24.5 (18–27)*	7.45 (7.41–7.48)	52.5 (49–57.3)	222.4 ± 34.5
48 h	93.5 (79.5–107.5)	87.7 ± 12.6	21.5 (16.8–26)	7.45 (7.41–7.48)	52 (49–56)	227.2 ± 40.5
*p* value^a^	0.083	0.097	0.000	0.884	0.542	0.002
HFNC (n = 44)						
Baseline	92 (80.5–107.8)	88 ± 6.0	18 (16–23)	7.48 (7.42–7.51)	50.5 (48–57.8)	239.2 ± 47.0
1 h	94.5 (86–103.8)	89.7 ± 7.9	21 (18–25)*	7.42 (7.40–7.48)*	56 (49.3–59)*	220.5 ± 36.3*
24 h	93 (80.3–99.8)	87.0 ± 8.8	20 (17.3–24.5)^#^	7.44 (7.41–7.48)	54 (49–58)	228.4 ± 35.3
48 h	92 (82.3–103.3)	86.1 ± 8.1	19 (16.3–23.8)	7.43 (7.41–7.49)	51 (49–57.8)	230.3 ± 36.0
*p* value^a^	0.454	0.129	0.009	0.002	0.016	0.000

Data are shown as means ± standard deviation or median (interquartile range)

*HFNC* high-flow nasal cannula oxygen therapy, *NIV* non-invasive ventilation, *HR* heart rate, *MAP* mean arterial pressure, *RR* respiratory rate, *PaCO*_*2*_ partial pressure of arterial carbon dioxide, *PaO*_*2*_ partial pressure of arterial oxygen, *FiO*_*2*_ fraction of inspiration oxygen

*Compared with the baseline value in the same group, *p* < 0.05 after Bonferroni correction

^#^Compared with NIV at the same time point, *p* < 0.05

^a^Comparison of multiple time points within the group

Arterial blood gas analyses showed that the PaO_2_/FiO_2_ and pH values in the HFNC group were lower than their baseline levels, while PaCO_2_ was higher than the baseline level 1 h after extubation (all *p* < 0.050, see Table 3). The PaO_2_/FiO_2_, pH, and PaCO_2_ in the HFNC and NIV groups 24 h and 48 h after extubation were not statistically different from the baseline levels.

### Other outcomes

**Fig. 3:**
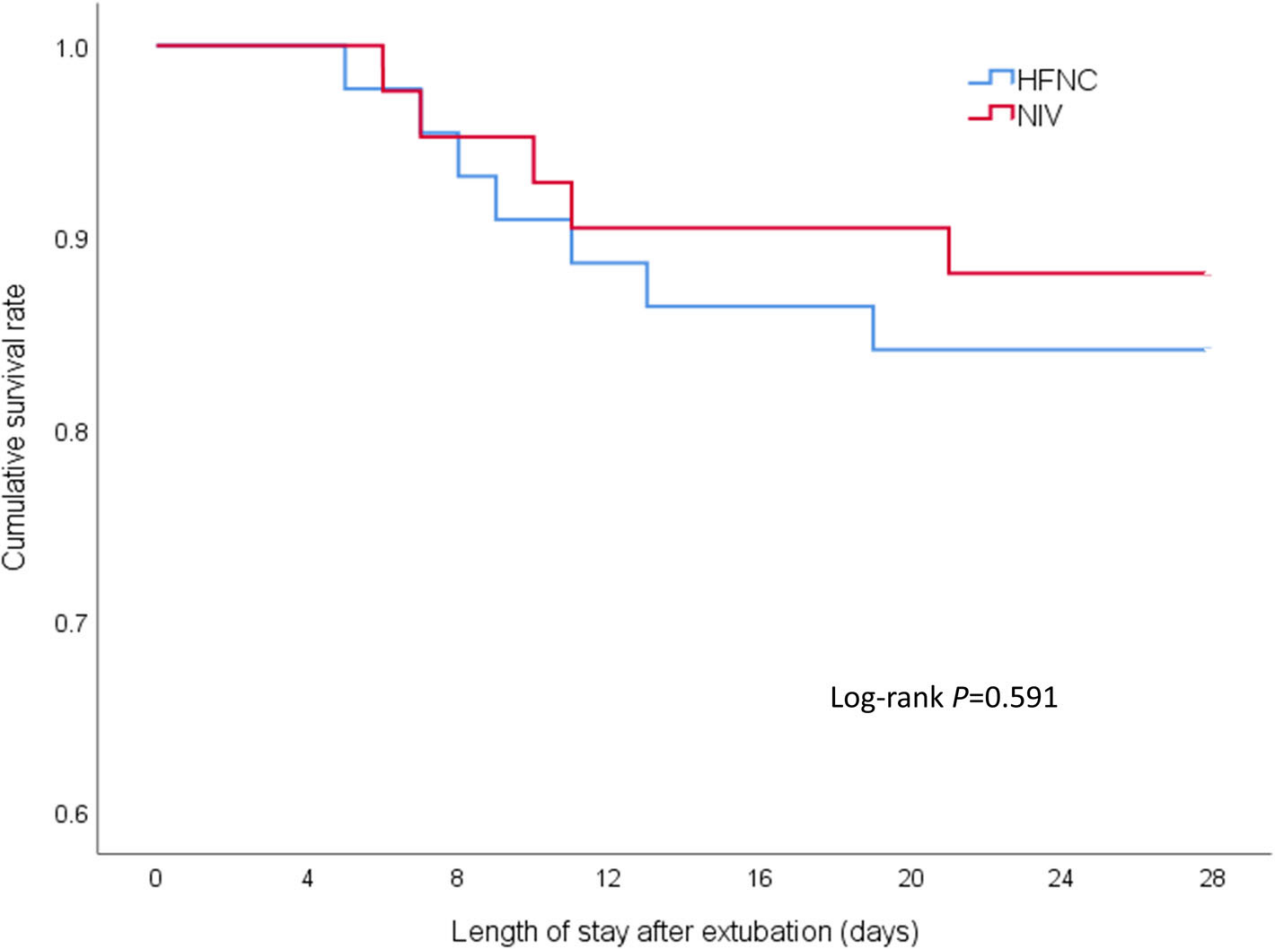
Kaplan-Meier curve analysis for cumulative survival rate. HFNC, high-flow nasal cannula oxygen therapy; NIV, non-invasive ventilation

There were no significant differences in the duration of post-extubation respiratory support, dyspnea scores, ICU, or hospital total lengths of stay between the two groups (all *p* < 0.050, see Table 4). The 28-day mortality in the HFNC group was 15.9%, which was not significantly different from the 11.9% in the NIV group (log-rank test 0.288, *p* = 0.591, see Fig. 3). The number of daily airway care interventions was significantly lower in the HFNC group than in the NIV group [6 (4–7) vs 7 (5–9.3), *p* = 0.006]. The comfort score in the HFNC group was also significantly higher than that in the NIV group [7 (6–8) vs 5 (4–7), *p <* 0.001], whereas the incidence of nasofacial skin breakdown was significantly lower in the HFNC group than in the NIV group (0 vs 9.6%, *p* = 0.027).

**Table 4:** Other outcomes in the HFNC and NIV groups

	HFNC (*n* = 44)	NIV (*n* = 42)	*p* value
Duration of HFNC or NIV, hours	83.9 ± 33.1	70.9 ± 30.6	0.063
Airway care interventions, per day	6 (4–7)	7 (5–9.3)	0.006
Comfort score	7 (6–8)	5 (4–7)	0.000
Dyspnea score	3 (2–4)	2 (2–3)	0.136
Nasal facial skin breakdown, *n* (%)	0 (0)	5 (9.6)	0.027
Length of stay in ICU, days	7.5 (6–10)	8.5 (6–12.3)	0.324
Length of stay in hospital, days	10 (8.3–12)	11 (9–14.3)	0.245
28-day mortality, *n* (%)	7 (15.9)	5 (11.9)	0.758

Data are shown as means ± standard deviation, number (%) patients, or median (interquartile range)

*HFNC* high-flow nasal cannula oxygen therapy, *NIV* non-invasive ventilation, *ICU* intensive care unit

## Discussion

This multicenter, randomized controlled trial showed that HFNC was not inferior to NIV at preventing post-extubation treatment failure and re-intubation for COPD patients recently extubated after hypercapnic respiratory failure. Compared with NIV, HFNC was more comfortable and better tolerated. The number of airway care interventions and the incidence of nasofacial skin breakdown associated with HFNC were significantly lower than in NIV. HFNC appears to be an effective means of respiratory support for COPD patients extubated after severe hypercapnic respiratory failure.

Invasive ventilation is sometimes necessary to rescue COPD patients with severe hypercapnic respiratory failure. Weaning strategies which include NIV are recommended as the standard treatment to reduce rates of ventilator-associated pneumonia and mortality without increasing the risk of re-intubation or weaning failure [22]. However, NIV intolerance appears in more than 15% patients due to various reasons, which increases the risk of treatment failure and re-intubation [23, 24]. Like in this study, many others have found that HFNC is often better tolerated than NIV, but data on COPD patients so far has been limited.

HFNC has been increasingly suggested for use in patients with COPD with acute hypercapnic respiratory failure. Bräunlich et al. reported that in 38 patients with an acute exacerbation of COPD and a pH of less than 7.38, HFNC increased the pH by 0.052 and reduced carbon dioxide by 9.1 mmHg [25]. In a prospective observational study involving 30 patients with moderate hypercapnic respiratory failure who were intolerant to NIV, patients’ pH improved and respiratory rate decreased with HFNC treatment, and the non-response rate to HFNC was only 13.3% [26]. Subsequently, two cohort studies with larger samples showed that for COPD patients with acute moderate hypercapnic respiratory failure, similar tracheal intubation and mortality rates were observed between HFNC and NIV, while HFNC was better tolerated [27, 28].

Other efforts to observe the efficacy of HFNC in COPD patients after invasive ventilation have been limited. In a cross-over study comparing HFNC to conventional low-flow oxygen therapy, HFNC was found to significantly decrease post-extubation work of breathing and neuroventilatory drive in COPD patients recovering from acute hypercapnic respiratory failure [29]. In a small randomized controlled trial, hypercapnic COPD patients received either HFNC or NIV immediately after extubation [15]. At 3 and 24 h after extubation, the pH in the HFNC group was higher than NIV group. No significant differences of vital signs and arterial blood gases were found at 48 h after extubation.

Unlike in the above study, the respiratory rate in both groups of our study increased at 1 h after extubation, which may be related to the relatively lower intensity of respiratory support after extubation. The respiratory rate in the HFNC group decreased to its baseline level 24 h after extubation, while the respiratory rate in the NIV group was still high at 24 h. This can be explained by the relatively poor tolerance of NIV and the increase in effective alveolar ventilation caused by the washout effect of dead space in HFNC.

One hour after extubation, the pH in the HFNC group of this study decreased and the PaCO_2_ increased, while the NIV group had no significant change from its baseline level. The difference between the two groups may be because HFNC does not have the added pressure support of NIV, resulting in decreased ventilation and oxygenation. However, the excellent tolerance and increased effective alveolar ventilation gradually made up for the above deficiencies, so that there was no significant difference in blood gas values between the two groups at 24 and 48 h after extubation.

To the best of our knowledge, this is the first randomized controlled trial to compare the failure rate of HFNC and NIV in patients with COPD after invasive ventilation. Treatment failure in this study was defined as reintubation or switch to the other treatment modality. Although the latter criterion added an element of patient subjectivity to the definition, this composite end-point reflects the pragmatic application of HFNC or NIV in everyday clinical practice [30]. Analysis of the causes of treatment failure in this study showed that treatment intolerance was significantly higher in the NIV group than in the HFNC group, suggesting that poor tolerance is an important reason for the failure of NIV treatment. Doshi et al. also found that 29% of NIV failures were attributed to treatment intolerance, which was significantly higher than the 4% rate of HFNC [31]. HFNC’s design does not lead to a sense of claustrophobia, which significantly improves compliance. At the same time, the heating and humidifying function of HFNC enables the gas delivered to reach an absolute humidity of 44 mg H_2_O/L and a temperature of 37 °C, which effectively promotes the discharge of secretions while avoiding side effects such as dry mucous membranes [32]. Because of these characteristics, patients can easily tolerate a gas flow rate of up to 50–60 L/min. The better tolerance of HFNC over NIV is clearly seen in comparing the comfort scores between the two groups.

The number of airway care interventions and cases of nasofacial skin breakdown in the HFNC group were also significantly lower than those in the NIV group, which was related to the HFNC nasal plug design and better comfort. Due to intolerance, drinking and eating, sputum clearance, communication, discomfort, or displacement of the NIV mask, NIV patients frequently remove their masks and significantly increase the nursing workload [28]. Patients in the HFNC group were not restricted by respiratory support in eating, drinking, and communicating. The incidence of skin breakdown and displacement of nasal prongs was extremely low.

There were some limitations to this study. First, the primary endpoint of this study was a composite of reintubation rate and switching to the other treatment modality, which has potential limitations described above. As for the re-intubation rate, the possibility of obtaining a positive result by increasing the sample size cannot be ruled out. Second, the settings for the HFNC gas flow in this study were based on each patient’s tolerance level, which is subjective. In subsequent studies, the HFNC gas flow could be titrated through diaphragmatic potential or ultrasound assessment of diaphragmatic muscle movement for better standardization. Finally, attending physicians could not be blinded to the study group since the devices were clearly different. However, investigators were excluded from clinical decisions and randomization was employed to help reduce bias.

## Conclusions

Among COPD patients with severe hypercapnic respiratory failure who received invasive ventilation, the use of HFNC as compared with NIV after extubation did not result in increased rates of treatment failure, while HFNC had better tolerance and comfort. These findings support the use of HFNC in such patients, especially for those who cannot tolerate NIV.

## Data Availability

The datasets used and analyzed during the current study are available from the corresponding author in response to reasonable requests.

## Abbreviations

COPD: Chronic obstructive pulmonary disease
NIV: Non-invasive ventilation
HFNC: High-flow nasal cannula oxygen therapy
PIC: Pulmonary infection control
ICU: Intensive care unit
APACHE II: Acute Physiological and Chronic Health Status Score II
SAPS II: Simplified Acute Physiology Score II
PaCO_2_: Partial pressure of arterial carbon dioxide
PaO_2_: Partial pressure of arterial oxygen
FiO_2_: Fraction of inspired oxygen
